# Rapid phylogenetic and functional classification of short genomic fragments with signature peptides

**DOI:** 10.1186/1756-0500-5-460

**Published:** 2012-08-28

**Authors:** Joel Berendzen, William J Bruno, Judith D Cohn, Nicolas W Hengartner, Cheryl R Kuske, Benjamin H McMahon, Murray A Wolinsky, Gary Xie

**Affiliations:** 1 Physics Division, MS D454, Los Alamos National Laboratory, Los Alamos, NM 87545, USA; 2 Theoretical Division, MS K710, Los Alamos National Laboratory, Los Alamos, NM 87545, USA; 3 Computer, Computational, and Statistical Sciences Division, MS B256, Los Alamos National Laboratory, Los Alamos, NM 87545, USA; 4 Bioscience Division, MS M888, Los Alamos National Laboratory, Los Alamos, NM 87545, USA

## Abstract

**Background:**

Classification is difficult for shotgun metagenomics data from environments such as soils, where the diversity of sequences is high and where reference sequences from close relatives may not exist. Approaches based on sequence-similarity scores must deal with the confounding effects that inheritance and functional pressures exert on the relation between scores and phylogenetic distance, while approaches based on sequence alignment and tree-building are typically limited to a small fraction of gene families. We describe an approach based on finding one or more exact matches between a read and a precomputed set of peptide 10-mers.

**Results:**

At even the largest phylogenetic distances, thousands of 10-mer peptide exact matches can be found between pairs of bacterial genomes. Genes that share one or more peptide 10-mers typically have high reciprocal BLAST scores. Among a set of 403 representative bacterial genomes, some 20 million 10-mer peptides were found to be shared. We assign each of these peptides as a signature of a particular node in a phylogenetic reference tree based on the RNA polymerase genes. We classify the phylogeny of a genomic fragment (e.g., read) at the most specific node on the reference tree that is consistent with the phylogeny of observed signature peptides it contains. Using both synthetic data from four newly-sequenced soil-bacterium genomes and ten real soil metagenomics data sets, we demonstrate a sensitivity and specificity comparable to that of the MEGAN metagenomics analysis package using BLASTX against the NR database. Phylogenetic and functional similarity metrics applied to real metagenomics data indicates a signal-to-noise ratio of approximately 400 for distinguishing among environments. Our method assigns ~6.6 Gbp/hr on a single CPU, compared with 25 kbp/hr for methods based on BLASTX against the NR database.

**Conclusions:**

Classification by exact matching against a precomputed list of signature peptides provides comparable results to existing techniques for reads longer than about 300 bp and does not degrade severely with shorter reads. Orders of magnitude faster than existing methods, the approach is suitable now for inclusion in analysis pipelines and appears to be extensible in several different directions.

## Background

As of this writing, DNA sequencers routinely produce more than 2 Gbp of data per hour, with the high-quality region of reads as short as 75 bp. Analytical methods that can keep up with this flow rate are urgently needed. Analysis is especially difficult for shotgun metagenomics data from environments such as soil where the diversity of sequences is high [1,2] and where sequences from close relatives are not to be found in reference databases (e.g. [3]). Insight into microbial communities and their dynamics would be desirable for a number of important applications in medicine, agriculture, ecology, and industry [4].

The first step in most sequence analyses is finding a suitable answer to the question, “How close is this sequence to something seen before?”. The notion of closeness implied in the question is a phylogenetic distance, which is most properly answered by a phylogenetic algorithm. Unfortunately the computational expense of such algorithms, coupled with the intractability of making the relevant alignments and trees for genes that may have large numbers of paralogs, make this approach infeasible at present except for a small fraction of gene families. The most common alternative is to find a proxy for phylogenetic distance in a more-readily-computed sequence similarity score as produced by the program BLAST [5] and its relatives. Yet the relationship between sequence similarity and phylogenetic distance is skewed by rates of acceptance of mutations that can range over many orders of magnitude over a length scale of tens of bases due to differences in functional constraints experienced by different parts of the gene [6]. Proteins from families of broadly-conserved genes and those parts of enzymes near an active site have significantly higher sequence identity than average [7]. The nature of current shotgun metagenomics data, with short reads from randomly-selected regions of genes, tends to accentuate the problem of transforming similarity scores to something resembling phylogenetic distances through injecting a noise term that can be difficult to remove by post-processing (e.g., [8-10]). This problem exists even for close matches, but is exacerbated as similarity declines since the underlying sequence alignment may also be called into question.

A variety of methods to classify shotgun metagenomic reads have been proposed, primarily based on protein families or gene clusters. These include partial assembly and hidden-Markov-model searches [11] of protein families [12,13]; finding the closest neighbors in either nucleotide or protein space using a variety of similarity scores [8,14]; and finding shared sub-strings of variable length via suffix trees [15]. Other alternatives to similarity scores include short-seed [16] and sub-HMM [17] methods. Phylogenetic analysis is typically the next step after classification, using Least Common Ancestor [8], nearest-neighbor [14,15], or hierarchical scoring [9] to assign phylogeny to the sequences identified in the classification step. Because of the numerous pitfalls in designing a computer algorithm to define functionally meaningful protein families [18], many classification pipelines require continual curation of protein families, which involves multiple-sequence alignment and the computation of a phylogenetic tree for each family, in the hope of identifying orthologous genes [12,19]. Such efforts are labor-intensive and limited by the paucity of biochemical validation of gene function. Another solution is to restrict analysis to a small number of well-behaved ‘housekeeping’ genes [20-22]. However, using this approach results in discarding the vast majority of sequence reads.

Exact amino acid *k*-mer matches with *k* in the range 3–6 have been employed to speed identification of homologous regions of genes for the purposes of constructing a multiple sequence alignment [23,24]. In this work, we are considering higher values of *k*, in order to identify homologous genes by comparing entire bacterial genomes. We begin by comparing the genomes of two divergent bacteria and observing that random matches dominate for *k* < 8, while for *k* = 10, an average of only about one random match is expected between the genomes. Such 10-mer matches are evidently long enough to specifically discriminate a portion of a conserved gene from other genes or organisms, while sufficiently short as to be present in both reference genomes and soil metagenomics data sets.

## Results

We begin by justifying our choice of *k* = 10 as the match length long enough to be specific, yet short enough to be prevalent in environmental samples. Building on this observation, we identify all 20 million strings of amino acids of length 10 which are shared by at least two reference genomes from distinct genera of bacteria. We denote these as orthogenomic signature peptides, and they serve as the foundation for the rest of our analysis. We then develop one algorithm to establish the correct phylogenetic placement of these signature peptides, another to classify metagenomic reads matched by signature peptides, and a final algorithm to functionally profile reads through use of an externally defined database. The use of fixed-length strings allows us to exploit standard index-based information retrieval techniques developed for web search engines.

### Choice of *k* = 10

**Figure 1:**
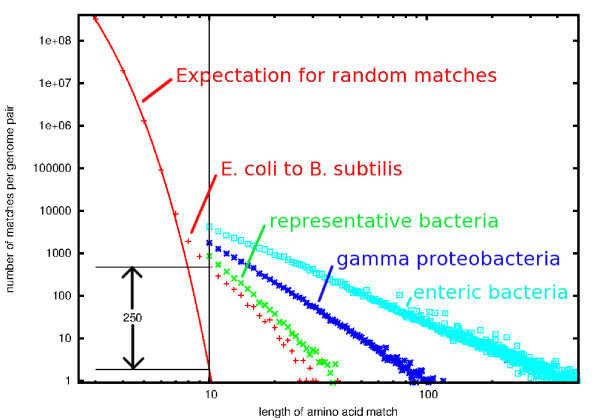
**Run-length distributions.** Symbols show the number of matches between *E. coli* and several sets of genomes as a function of the length of the exact amino-acid match. Each match is counted only once, at the value of its maximal extension. For *E. coli* compared to *B. subtilis* (red crosses), the distribution is extended down to *k* = 3, and an exponential fit is shown as a solid red line. For *k* > 9, run-length distributions are shown for *E. coli* compared to a set of 22 other representative bacteria (green x), a set of 35 gamma proteobacteria (blue asterisks), and 17 representative enteric bacteria (cyan boxes).

Figure 1 shows the run-length distribution of shared amino acid *k*-mers between *Escherichia coli* and various sets of bacterial genomes, for *k* in the range of 3 to 500. It is dominated by random matches for *k* < 8, and dominated by gene matches for *k* > 8. The solid red line is an exponential fit to the run-length distribution of amino acid matches between *E. coli* and *Bacillus subtilis*, reflecting a 16-fold reduction in the number of matches for each increase by one in the run-length, *k*. The number of random matches quickly drops with increasing *k*, reaching 1.8 per pair of bacterial genomes for *k =* 10, with a ratio of observed matches of length 10 to the expected number of random matches of 250. Since the rest of our analysis will treat matches longer than 10 as multiple overlapping 10-mers, and the histogram in Figure 1 counts matches only once, at the full extent of their match length, the appropriate ratio of non-random to random matches is not 250, but 1000.

In addition to the small-*k* behavior of random matches, Figure 1 shows the large-*k* behavior for *E. coli* scanned against three sets of bacteria. As the phylogenetic distances decrease, both the number and length of amino acid matches increase greatly. The frequency distribution of peptide match lengths is a power-law distribution, indicative of the broad diversity of functional pressures on proteins rather than the 20-fold falloff one naively expects from random matches when the match-length is increased by one amino acid. Interestingly, our choice of 10 residues is only slightly longer than the average length of epitopes recognized by the mammalian immune system.

### Signature occurrence

**Figure 2:**
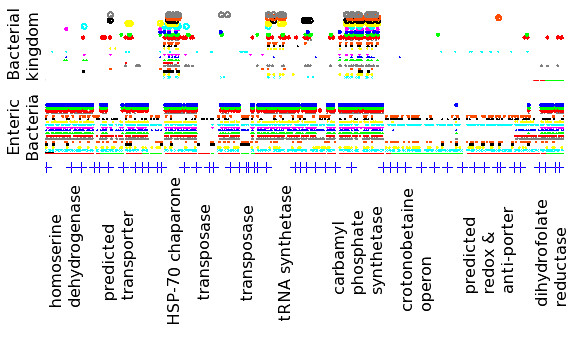
**Distribution of matches of length 10 or longer across the first 50 kilobases of the*E. coli*genome.** Starts of individual genes are indicated by the blue crosses along the bottom, and matches to a particular genome are indicated in a line above the crosses in a particular color. The first line above the crosses indicates the coding direction of the proteins: either forward (red) or reverse (green). Names of several genes and operons are indicated at the bottom. Black squares in the bottom portion and cyan triangles in the top portion indicate matches to other portions of the *E. coli* genome (paralogs) are shown for completeness, but not discussed further in this work. The matching signature peptides and annotations of matched genes are enumerated for *B. subtilis* (black squares in the top panel) in Additional file 1, and discussed below.

Figure 2 shows the *k*-mer matches of length 10 or greater along the first 50 kilobases of the *E. coli* genome to 45 other genomes at varying phylogenetic distance from *E. coli*. The comparison of *E. coli* to other enteric bacteria, at the bottom of Figure 2, shows that each of the first 47 genes contains a match to at least one other genome, and most genes contain matches throughout. From the comparison to representative genomes across the bacterial kingdom, at the top of Figure 2, most of the matches are shown to occur to such recognizable genes as heat shock protein 70, carbamyl phosphate synthetase, and a tRNA synthetase. Inspection of the multiple sequence alignments made of matched genes (data not shown), and annotation of the genes matched by the 10-mers shows that the identified sequence homologies extend beyond the match. The agreement in annotated function is generally evident, although often somewhat vague.

A vertical slice through Figure 2, then, will approximate the presence / absence phylogenetic profile of each gene across enteric bacteria (bottom portion) and representatives of the bacterial kingdom (top portion). Three types of matches can be distinguished by the 10-mer-based phylogenetic profile. Highly conserved proteins, such as HSP-70 or the tRNA synthetase, have 10-mer matches between *E. coli* and each of the other genomes presented in Figure 2. In this case, multiple sequence alignments can be made across the bacterial kingdom, and the 10-mers, when examined across all of the pairwise bacterial genomic comparisons, serve as an enumeration of all of the different ways each conserved region can be assembled. Quite frequently in this case, some 10-mer signatures are indicative of function across the entire bacterial kingdom and will be useful in identifying divergent organisms in metagenomic samples, while other 10-mer signatures identify the gene in only a particular phylogenetic subset of bacteria, and will be useful in creating a phylogenetic profile of a metagenomic sample.

Other genes, such as the crotonobetaine operon [25] and predicted redox and antiporter genes in Figure 2, are present in only a few of the representative genomes, but have matches throughout the operon. These genes are likely to have a related function because they are colocalized on the genome and are only present in a specific subset of genomes; the particular nature of the phylogenetic profile could be used to associate genes with one another or with metabolic strategies, such as ammonia oxidizing or pathogenicity, of the bacteria in which they are found.

Finally, matches are observed in Figure 2 where only a portion of a gene is conserved, but that portion is conserved across much of the bacterial kingdom. One of the most common instances of this case is the ATP binding domain of transporter proteins, where this energy transduction domain is highly conserved, while the region determining substrate specificity is highly variable. Such domains frequently involve matches to numerous paralogous genes.

### Signature specificity

**Figure 3:**
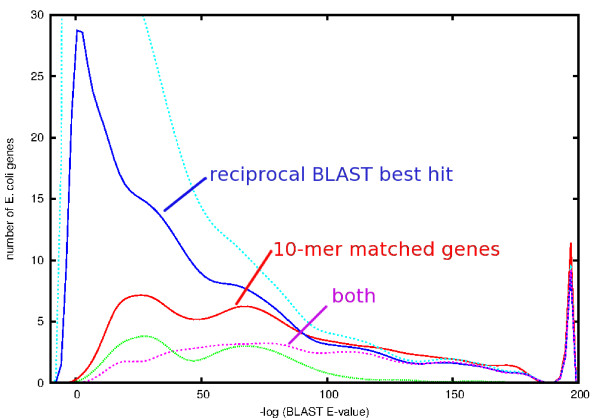
**Distribution of Protein BLAST scores (−log(E-value)) for various sets of*E. coli*genes scored against genes in the*B. subtilis*genome.** At the top, in cyan, is the distribution of the best-match BLAST scores for each of the 4145 genes in the *E. coli* genome. 1461 of these are also reciprocal best hits of the *B. subtilis* genome against *E. coli*; the distribution of these scores is shown in dark blue. 746 distinct pairs of *E. coli* – *B. subtilis* genes are connected by one or more 10-mer matches; the distribution of BLAST scores for these matches is shown in red. In magenta is shown the distribution of BLAST scores for the 388 genes that are both reciprocal BLAST best hits and connected by one or more 10-mers. At the bottom of the plot, in green, is the distribution for genes with matching 10-mers and the word ‘transporter’ in either gene’s annotation. The peak at the right of the plot indicates the 37 pairs of genes given an E-value of ‘0.0’ by BLAST.

The specificity of 10-mer matches is assessed in Figure 3, which shows the distribution of protein BLAST scores (−log(E-value)) for various sets of *E. coli* genes scored against genes from the *B. subtilis* genome matched in various ways. For BLAST E-values more significant than ~10-^100^, all algorithms return a similar set of 210 highly-conserved genes, including the RNA polymerase, several tRNA synthases, nitrate reductase, and DNA gyrase. For matches with less significant E-values, not only do the reciprocal BLAST best-hits return far more matches with lower BLAST scores than do the signature-matched genes, but the signatures also return multiple matches for each *E. coli* gene. Examination of the annotation of these matches reveals that paralogs such as transporters and transcription factors comprise the bulk of the low-specificity matches. These are the genes most likely to differ in inventory across genomes, and thus complicate both functional and phylogenetic assignments [26].

To understand the least-significant matches better, we examined the ten pairs of genes with a 10-mer match between *E. coli* and *B. subtilis* having a BLAST E-value less significant than 10-^10^. Examination of the respective annotation reveals only two pairs of genes with inconsistent annotation. One of these pairs also matches at four of the six amino acid positions immediately before the signature match, and is labeled ‘hypothetical protein,’ perhaps implying the decay of a duplicated gene. Five of the matches are to ATP-ase domains of transporter proteins. Together, these five genes match 618 genes in *B. subtilis* with a BLAST E-value better than 0.001, while only 28 genes in *B. subtilis* are matched by these five genes with an amino acid 10-mer. In only one case were we unable to discern why the match occurred.

Typical bacterial proteomes contain about 10^6^ amino acids, so the likelihood of finding a 10-mer match by searching one genome against another, purely by chance is ~ 20-^10^ x (10^6^)2, or approximately 10%. The non-uniform occurrence of amino acids increases this estimate somewhat; i.e. using the frequency of each amino acid in *E. coli*, the most likely 10-mer is AAAAAAAAAA, which would occur randomly at one part in 6x10^11^, while the 10-mer at the core of the RNA polymerase, GGQRFGEMEV would occur randomly at twice the rate estimated from a uniform distribution of amino acid usage, i.e. two parts in 10^13^. Empirically, from the exponential fit in Figure 1, a fall-off of 16-fold in the number of matches for each increase by one occurs, as *k* increases from 3 to 7, is observed, producing a randomly-occurring rate of 10-^12^. As a further test of the specificity of 10-mer matches, we identified only four 10-mer matches between the five incorrect reading frames of each *E. coli* gene and the complete proteome of *B. subtilis*, with one 10-mer, STSSSSSSSS, occurring twice.

Four independent calculations (the estimate above, the estimate from Figure 1, examination of gene pairs with the worst BLAST scores, and searching incorrect reading frames against a proteome) all suggest that random matches account for approximately one match out of the one thousand ‘correct’ matches when comparing two divergent bacterial proteomes such as *E. coli* to *B. subtilis*. We chose two well-annotated and reasonably divergent genomes to assess the specificity of a single 10-mer amino acid match between genomes, but we expect the likelihood of random matches to depend on the quantity of protein sequence compared, not the source. When analyzing metagenomic reads with signature peptides, this specificity will be unaffected by fragment length, all the way down to the 30 nucleotides necessary to encode 10 contiguous amino acids. BLAST specificity, however, will suffer greatly at short read lengths, due to the global nature of the similarity score, as well as the absence of accurately called start sites within short metagenomic reads, as characterized in [27].

In supporting online Additional file 1, we enumerate all 1,030 matches of 10 or more contiguous amino acids shared between *E. coli* and *B. subtilis*. We invite the reader to use 10-mer, or even 6-mer, match strings from this dataset to search the database of complete genomes, comparing annotations and aligning the sequences of the genes returned. Sequence homologies of the genes extend well beyond the single 10-mer matches, and the agreement of annotations between matching genes is readily apparent. In many cases, such as the RNA polymerase or pyruvate kinase, the annotated function is identical in the two organisms. In the case of ABC transporters or response regulators, however, some signatures are generally indicative of the protein family, while others distinguish particular types of ABC transporters or response regulators.

The high prevalence of ATP-ase domains of transporter proteins in the set of gene pairs with matching strings is striking when scanning Additional file 1, and we confirm their importance by including in Figure 3 the BLAST scores for 10-mer matches between *E. coli* and *B. subtilis* with the word ‘transporter’ in the annotation of either gene. 87 transporter genes in *E. coli* share one or more 10-mers with *B. subtilis* genes, producing a total of 252 distinct pairs of matched genes, or one third of the total number of matched pairs. Together with other paralogous genes, they make up the half of the matches shown in Figure 3 with an E-value less significant that 10-^100^.

**Figure 4:**
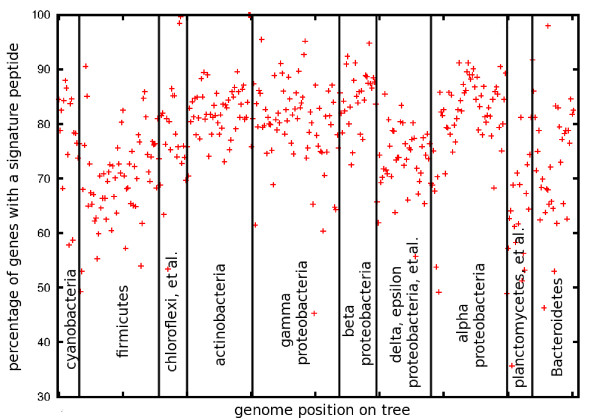
**Fraction of genes containing at least one signature peptide in genomes across the 403 bacterial reference genomes.** As described in the text, signature peptides are exact matches of length 10 between genomes of different bacterial genera. The genomes are ordered along the x-axis according to their position in our bacterial phylogeny provided as Additional file 2 and Additional file 3; the ordering corresponds to that in Figure 5, starting at the 9:00 position and proceeding counter-clockwise around the radial tree.

The list of signature peptides generated by identifying all 10-mers matching across genera in our dataset of 403 reference genomes numbers 20 million, reflecting 5% of the total number of 10-mers in those genomes. This list hits an average of 77% of the genes in our one-per-genus reference set, with considerable variability in coverage among genomes. Figure 4 shows the profile of the fraction of genes containing a signature peptide as a function of the phylogenetic placement of each organism. Divergent organisms, such as *Gemmatimonas aurantiaca* or *Elusimicrobium minutum*, contain signatures to most of their genes (74% and 62%, respectively), while many of the proteobacteria contain signatures in more than 90% of their genes. The firmicutes and planctomycetes are relatively under-representated in this respect. While the ATP-ase domains of transporters dominate the peptide signatures at large phylogenetic distances, most genes and gene families are eventually identified in genomic comparisons as the phylogenetic distance decreases, consistent with the behavior shown in Figure 2.

### Phylogenetic placement of signature peptides

**Figure 5:**
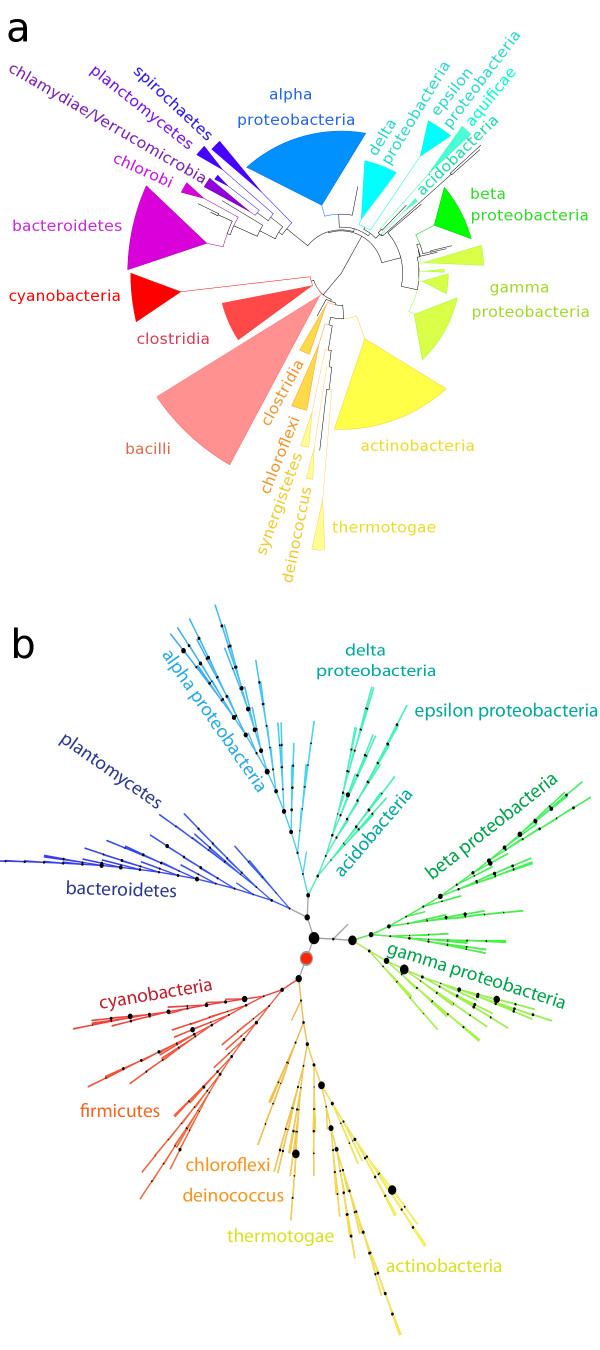
**Bacterial phylogeny, and the distribution of 20 million orthogenomic signatures across this phylogeny.** ( **a**) Our computed RNA polymerase based phylogeny, showing the deep branches between bacterial phyla, and ( **b**) The distribution of signature peptides across the nodes of this phylogeny, with branch-length information removed. The symbol area at each node represents the fraction of total number of signatures assigned to the node. The root node, with 11% of the signatures, is shown in red. Most phyla are labeled and can be used together with the complete tree (Additional file 2, Additional file 3) to identify which taxa are covered by each node.

In order to provide an accurate reference phylogeny for the signature-placement and read-placement algorithms, we chose to compute a tree from the concatenated sequences of the beta and beta prime subunits of the RNA polymerase of each reference bacterium. Use of this gene for phylogenetic inference is considered superior than other markers because of its high information content and its central location in the regulatory pathways involving the bacterial transcription apparatus [28]. Considerable effort was expended to align the sequences accurately, to mask regions that would be inappropriate to include in the tree-building model, and to use a maximum-likelihood tree building method in conjunction with an evolutionary model based on functional pressure. Details are provided in the Methods section. A simplified version of the tree is presented in Figure 5 with a more detailed version provided in pdf and phyloxml formats in Additional file 2 and Additional file 3. Although more detailed than the NCBI taxonomy, general agreement was observed between their classification and ours. The behavior of a number of deeply branching roots, visible in Figure 5, shows differences when compared to other treatments, but we expect these differences to have minimal impact on the results presented.

In our analysis, we treat a match of length 50 as 41 overlapping 10-mer signature peptides. Consequently, we need to create algorithms to analyze the frequent case of multiple signature matches being present in a single metagenomic read. We chose to first assign signature peptides individually to nodes on the phylogenetic tree. The goal of the signature-placement algorithm is to provide the appropriate degree of phylogenetic specificity for each signature. We use the conservative least common ancestor [11] algorithm for this purpose. Each signature is placed on the tree at the most specific node covering all leaves at which the signature was observed. Details and a diagram of this process are shown in the Methods section. Figure 5b shows the distribution of the 20 million signature peptides across the nodes of the bacterial phylogeny. The most populated node is at the root, with 11% of the signatures. However, an ample supply of signatures is found throughout the tree. In addition, more highly populated nodes are often found in well-delineated clusters, typically corresponding to well-known phylogenetic divisions (e.g., cyanobacteria or enteric bacteria).

Phylogenetic assignment can be confounded by the ubiquitous processes of gene duplication [29], domain swapping [30], and horizontal gene transfer [31,32], as well as the differing gene inventories among bacteria [26,33]. By choosing a signature-by-signature placement on the phylogenetic tree, we are eliminating the ortholog identification steps from the phylogenetic profiling process. In essence, our approach replaces the question of gene sequence similarity with 'Where have the signature peptides been seen before?'. Since some signatures appear in dozens of reference genomes, our decision to place the signature peptide far enough towards the root of the tree to cover every observed instance of the signature can be seen as a conservative choice. A specific phylogenetic assignment will only be made if no conflicting evidence is available, so observed phylogenetic signals reflect the self-consistency of our assumptions.

Another consequence of our signature placement algorithm is that both functionally constrained signature peptides from divergent bacteria and 10-mers derived from horizontally transferred genes are placed near the root of the tree. It seems likely to us that further analysis of the phylogenetic density of signatures could algorithmically distinguish between these two cases, but we do not attempt that here.

### Phylogenetic classification of metagenomic reads

We classify the phylogeny of metagenomic reads with a second algorithm, called the greatest common descendent algorithm, which is described in detail in the Methods section, below. For reads with one or more signatures assigned to a particular node, the read is assigned to that node. For reads matching signatures from nodes in a path from the root towards a particular leaf, the read is assigned to the most specific node (closest to the leaf) along that path. If that path branches, the read is assigned to the branch-point. For the case of overlapping signatures, this algorithm is typically equivalent to using the full-length match as a signature peptide. Like the signature-placement algorithm, it is designed to be conservative, in the sense that phylogenetic assignments will be as specific as possible, provided that no conflicting evidence is present.

Although we have shown that individual signatures are both specific and plentiful, the sensitivity and specificity of the overall read-placement process is difficult to estimate analytically. We first verified that two organisms in our reference database, *Elusimicrobium minutum* and *E. coli*, are correctly classified (the first at the root, the second along a path from the root to the most specific node covering *E. coli*) by treating raw sequencing data as a metagenomics data set (data not shown). It is a necessary consequence of our signature-placement and read-placement algorithms that every assigned read will be placed along the path from that organism to the root of the tree.

To assess the maximal likely extent of database representation bias on sensitivity for novel organisms, we generated synthetic metagenomic reads from the finished sequence of two bacterial genomes from genera not in our database, representing two extreme cases. *Shigella flexneri* is phylogenetically close to both *E. coli* and Salmonella enterica, while the other genome, *Dehalogenimonas lykanthroporepellens*, is from a deeply-branched genus in the phylum chloroflexi, with no close neighbors among our set of reference genomes. In both cases, overall phylogenetic assignment of the synthetic reads was appropriate, with *S. flexneri* reads assigned overwhelmingly (85%) to the most specific node covering *E. coli* and S. enterica, while *D. lykanthroporepellens* reads were for the most part assigned to the root node; only a small, but significant, portion was assigned to the chloroflexi phylum. For *D. lykanthroporepellens*, the sensitivity was proportional to read length, as expected for a local signature-based method far from saturation. For *S. flexneri*, the sensitivity approaches the limit given by the fraction of the genome coding for proteins. The ratio of sensitivities, a measure of the database bias, was a factor of five for 75-bp reads and decreased to less than a factor of two for 600-bp reads. It should be possible to decrease this database bias by utilizing a subset of the signatures and incorporating more reference genomes.

In order to make as direct of a comparison as possible with other methods on novel bacterial genomes of relevance to soil microbiology, we created synthetic data of fixed read lengths 75, 150, 300, and 600 base pairs and no synthetic errors added, from each of four draft genomes (99% complete) cultured from a desert soil consortium. These data were analyzed with our signature peptides, with MEGAN analysis of against both the NR and NT databases downloaded on 14 February, 2012, and a protein BLASTX against a database of the same 403 bacterial genomes used to generate our 20 million signature peptides. These comparisons are meant to be a representative sample of the types of analysis presently in common use. A tar file with all sixteen synthetic data sets is provided as Additional file 4.

**Figure 6:**
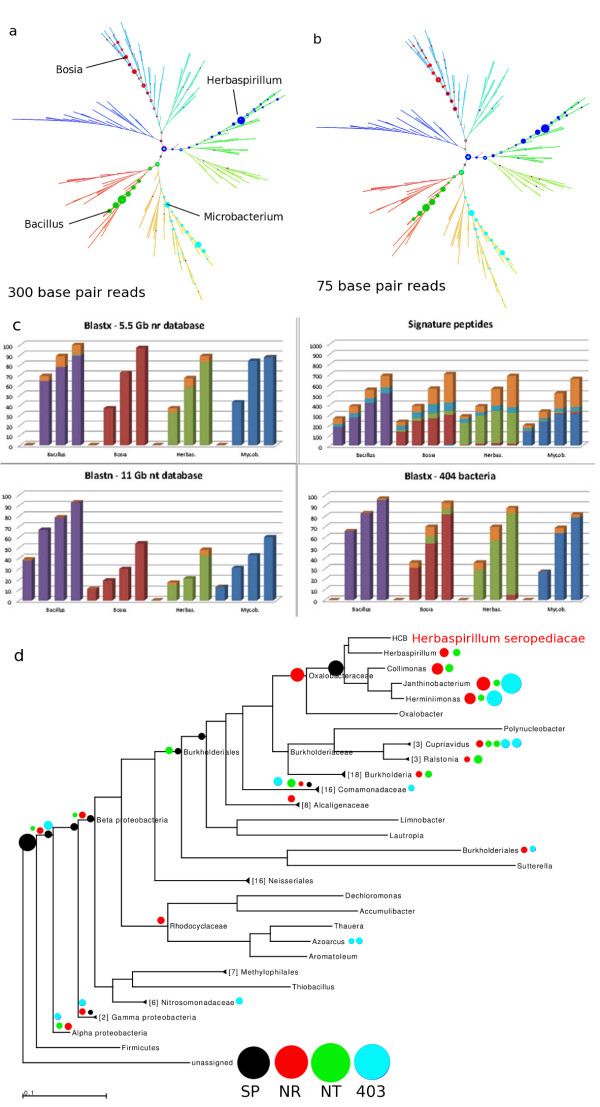
**Sensitivity and specificity of simulated reads from draft soil genomes.** Simulated reads were constructed using MetaSim [63] from genomes of four soil bacteria. *Herbaspirillum seropedicae* and *Bacillus mojavensis* are species from genera represented in the BLAST databases NR and NT as well as our signature peptide database (SP). *Microbacterium trichotecenolytcum* represents a genus found in NR and NT but not in SP. *Bosea thiooxidans* is from a genus not found in any of the three. ( **a**) Specificity of placement of simulated reads on the reference tree using our method, for 300-bp reads. ( **b**) Placement of 75-bp reads using our method. ( **c**) Comparison of sensitivity of our method (top right panel) and MEGAN [8] using three different BLAST databases: BLASTX and NR (top left) BLASTN and NT (bottom left), and BLASTX against the same genomes used in SP (bottom right). Simulated read lengths of 75, 150, 300, and 600 bp were used for each of the four genomes in each of the four panels. Colors indicate specificity of placement, with gold indicating non-specific placement near the root node in each case. (**d**) Details of specificity of placement of simulated 150-bp Herbaspirillium seropediacae for the 4 methods: our method (black), MEGAN4 with BLASTX against NR (red), MEGAN4 with BLASTN against NT (green), and MEGAN4 with BLASTX against the same genomes used in SP (cyan).

**Figure 7:**
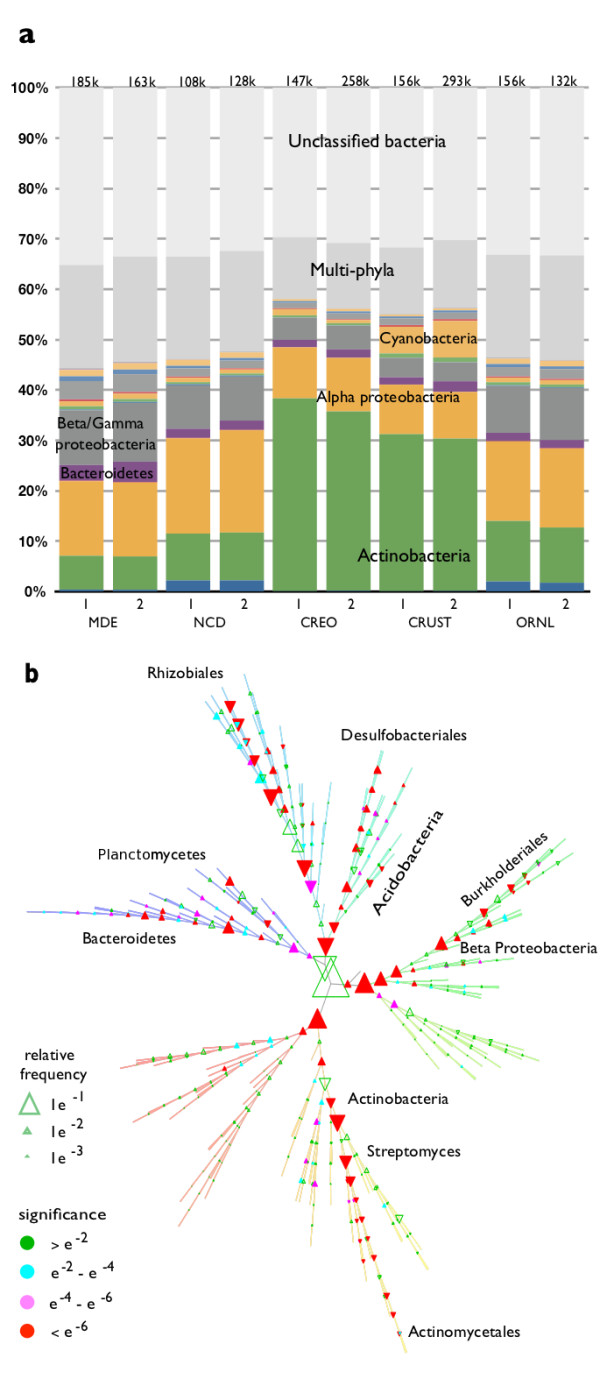
**Phylogenetic breakdown of metagenomic soil samples.** ( **a**) shows the high level classification of the metagenomics reads across 10 samples (two field replicates from each of 5 different sites), with the number of reads identified as bacterial at the top of each column, in thousands. ( **b**) shows the differences between samples from the MDE and NCD sites across all 402 interior nodes of the phylogenetic tree. Symbol size indicates number of recruited reads, while color indicates the statistical significance of the change (p-values: blue ~0.05, red ~0.000001). Triangles which point up indicates a higher prevalence in MDE; triangles with point down indicates a higher prevalence in NCD.

Signature-peptide-based profiles of the four genomes are shown in different colors in Figures 6a for 300 base pair reads and Figure 6b for 75 base pair reads, with the same layout as in Figures 5 and 7. The correct placement of each genome, according to placement in an RNA polymerase beta–beta prime based tree, is indicated in each panel, with both *Herbaspirillum seropedicae* and *Bacillus mojavensis* representing novel species of genera already represented in our reference database, *Microbacterium trichotecenolyticum* representing a genus in Genbank, but not our reference database, and *Bosea thiooxidans* representing genus novel to both NR and the signature peptides. For the two organisms with nearer neighbors in our reference database, most of the population is assigned to only one or two specific nodes, with almost nothing either on a wrong branch or overly-specific. For the two more-novel organisms, *Bosia* and *Microbacterium*, the populated nodes are more spread out, although still predominantly between the root and the correct placement on the tree. Only minor differences are found when 75-base-pair reads are searched, compared to 300-base-pair reads.

The sensitivity of the signature peptides and four BLAST-based methods are compared in Figures 6c, with the phylum of assignment indicated on the respective bar graphs. All methods correctly classified the phylum of all of the reads, but a significant fraction of the reads did sometimes get assigned to the root. That the signature peptides would have a slightly lower sensitivity than the two BLASTX methods might be anticipated from the fact that only approximately 80% of the genes contain signature peptides, while BLASTX utilizes all genes. It should be noted that the two attributes that cause a gene to not contain a signature peptide, low conservation of sequence or frequent absence from neighboring genomes, both make the gene less suitable as a phylogenetic marker. The signature peptides also show a minimal database bias (all four genomes show the same amplitude) and read-length bias (a linear increase in sensitivity with read-length, reflecting the greater chance of observing a signature peptide). Relatively little difference in overall sensitivity was observed when comparing protein BLAST against the 403 reference genomes and NR. The nucleotide BLAST showed both the greatest database bias and lowest sensitivity for three of the genomes. Both protein-BLAST based methods were unable to place any 75-base-pair reads. BLAST was run with an E-value cutoff of 10-^10^ and MEGAN employed a default cutoff of 35 for the bitscore and a requirement of 10% similarity to the top hit for consideration of alternative matches.

We investigated the specificity of read assignment in some detail in Figure 6d, which shows read-placements on an RNA polymerase-based tree for *Herbaspirillum seropedicae* synthetic data, totaled across the four read-lengths. The biggest difference was the expected propensity of BLAST and MEGAN to place over-specifically, as has been noted before [9,10]. Somewhat more breadth in phylogenetic placement results from using a BLAST database of only the 403 representative bacterial genomes, instead of NR.

### Throughput

Both absolute and relative run-times of sequence analysis methods will depend, sometimes substantially, on the type of data being analyzed and the hardware and operating system being used for analysis. Nevertheless, we feel it is important to benchmark the various methods on a particular data set, made available as Additional file 4, on a single CPU of a desktop machine (Intel i7 processor, 6 GB RAM, under $1000). For the BLAST-based method, timings were based on the first 100 reads of each file in Additional file 4, and resulted in an average throughput of 25 kbp/hr for BLASTX against NR, 402 kbp/hr for BLASTX against 403 genomes, and 1.1 Mbp/hr for BLASTN against NT. The observed rates were roughly linear in the total number of base pairs analyzed when the length of the synthetic reads was varied, and varied somewhat across the different organisms. Even on the full 1000 reads of the files in Additional file 4, the signature peptide method was fast enough to be difficult to time, so we simply re-ran all of the FACE site metagenomics data (1.7 Gbp) in under 15 minutes of clock time on a single processor. Specifically, analysis required 30 seconds to read the signature peptide data file and subsequently processed the reads at a rate of 6.6 Gbp/hr. Roughly 60% of the analysis time was spent translating the reads and 40% in matching to the signature peptides.

### Effect of changing *k*

It is illustrative to examine what happens when we repeat our overall process for the case of *k*-mers of length *k* = 8, where from Figure 1, we estimate the number of random matches has increased by a factor of 256, while the number of matches indicating homologous genes has risen by only a factor of five. For *k*-mers of length *k* = 8, matches were found throughout the bacterial phylogeny, indicating both specific and root-level signatures. When challenged with metagenomic data, however, more than 90% of the reads were assigned to the root of the tree, because of conflicting specific assignments (data not shown). Evidently, the signatures assigned near the leaves of the tree either lacked the generality necessary to be found in metagenomic sequences not in the reference database, or were placed there because of insufficient sampling of the reference. In either case, it serves as an important check on our process, that in cases of ambiguity, metagenomic reads will be placed at or near the root of the tree. For similar reasons, mistakes or ambiguities in the assumed phylogeny of bacterial organisms will also result in a greater fraction of metagenomic reads being assigned to the root of the tree, and not in a false precision in read assignment.

The case of larger *k* will decrease the number of signatures, as evident from the run-length distributions shown in Figure 1. While the specificity of individual signatures will be higher, this specificity is largely captured with the read-placement algorithm and overlapping 10-mers. An estimate of the decreased sensitivity that would occur by increasing *k* to 11 can be made by observing that only 17% of the soil metagenomic reads contained only one signature and by assuming that the majority of multi-signature reads occur from overlapping signatures. This relatively small decrease in sensitivity is in keeping with the relatively small decrease in the run-length near distributions near *k* = 11 in Figure 1, compared to the 16-fold decrease in the number of random matches.

### Phylogenetic profiles of soil metagenomes

In order to characterize how our method performs on real data, we examined shotgun metagenomic reads from ten soil samples collected at five different ecological sites from the Free-Air Carbon Enrichment (FACE) project [34]. The two samples from each site function as field replicates in our analysis. The sites include an estuary in Maryland (MDE), a deciduous forest in North Carolina (NCD), a bacterial-mat crust (CRUST) in the Nevada desert, together with a nearby patch soil partially shaded by creosote bush (Larrea tridentata) (CREO), and a tree plantation in Tennessee (ORNL). Details of the sample collection, preparation, and sequencing are provided in the Methods section. We analyzed a total of 4.4 million metagenomic reads with an average length of 383 base pairs. On average, 39% of the reads across the ten samples could be identified as bacterial and placed on the tree by our method.

Figure 7a provides the rolled-up phylum-level view of the composition the ten samples, with the number of reads identified in each sample indicated at the top of each bar (in thousands). As expected, differences between field replicates at the same location are much smaller than differences among different locations. It is also noticeable that the two desert sites (CRUST, CREO) and the two forest sites (NCD, ORNL) appear to have distinctive phylogenetic profiles. Approximately half of the reads containing a signature peptide are not classified to a single phylum. Part of this is due to highly-divergent bacteria.

Figure 7b compares the phylogenetic profile of the MDE sample to the NCD sample across all 403 internal nodes of our reference tree, using the same layout and branch colors as Figure 5b. Several well-known families of soil bacteria are observed, and these are indicated by labels in Figure 7b. A common pattern in Figure 7b is to find reads assigned along a line from the root to one particular genus-level node at the tip of a branch. This type of pattern could arise either from the presence of a broad range of species of varying phylogenetic distance to a reference genome (and thus a varying mix of highly conserved and more specific signature peptides) or because of an intrinsic blurriness to our analysis method. The plausibility of the data shown in Figure 7 is supported by the significant and repeatable information content, and the existence of similar phylogenetic profiles for comparable ecosystems. In addition, the synthetic data, representing single genomes, in Figure 6 shows a relatively sharp placement on the tree, while close inspection of Figure 7 shows numerous instances of nearby nodes changing in opposite directions.

83% of the matching reads contained more than one signature, allowing for another self-consistency check. Of the reads with multiple signatures, 19% contained signatures assigned to a single node of the tree while 55% had signatures assigned to multiple nodes from a single hierarchy (monophyletic). The remaining 26% of the multiple-signature reads were associated with multiple nodes from multiple hierarchies (polyphyletic), indicating conflicting phylogenetic assignments. As described earlier, such reads were assigned to the most specific node covering all conflicting assignments, often near the root of the tree.

We provide a table of the number of reads recruited to each of the nodes of the phylogenetic tree for each of the ten samples, Additional file 5. Node numbers provided in this table correspond to those on the nodes of the tree provided as Additional file 2 and Additional file 3. Many additional experimental techniques were applied to these FACE sites, with results to be published elsewhere.

### Accuracy of phylogenetic read assignments

Although our results in Figure 7 are plausible and pass several self-consistency checks, we attempt here to assess the accuracy of phylogenetic read assignments. Table1 compares selected statistics from our method for the CREO site versus three established analysis methods for shotgun metagenomic reads, together with the results of a 16S rRNA survey performed on similar samples via saturation PCR followed by Sanger sequencing. While the numerical differences in populations identified by the different methods varies by a factor of two or more, in all three phyla shown, the five methods were typically repeatable in field replicates to within a few percent, with all methods agreeing upon the sign of the change in every case. The differences among phylogenetic profiles from the four shotgun metagenomic data analysis methods are as large as the differences between the various shotgun analyses and the rRNA survey.

**Table 1:** Comparison of methods applied to two metagenomic data sets

	**This work**	**MG-RAST**	**MEGAN**	**AMPHORA**	**16S rRNA**
Average % reads ID’d as bacteria	46	49	63	0.3	
Average % bacterial ID’d as actinobacteria	36.9	49.2	42.6	45.1	28.4
Difference (CREO2 -1)	−2.6	−3.6	−3.5	−2.2	−5.1
Average % bacterial ID’d as α-proteobacteria	10.4	20.8	15.1	35.1	29.4
Difference (CREO2 -1)	+0.5	+0.9	+0.7	+1.1	+4.0
Average % ID’d as cyanobacteria	0.9	2.6	1.6	0.5	4.0
Difference (CREO2 - 1)	−0.6	−0.8	−0.9	−0.8	−2

Selected populations and their changes with field replicates from the desert creosote site (CREO1 and CREO2) are shown. An average of 448k unique reads with an average read length of 375 base pairs was analyzed per sample. Default BLAST E-value cutoffs of 10-^3^ for MEGAN, and 10-^5^ for AMPHORA were used. For MG-RAST (v2) we used a E-value cutoff of 10-^10^, tighter than the default, because it resulted in a similar fraction of reads being assigned. Normalization and definitions of unique reads differ somewhat among methods.

The numerous sources of bias in obtaining phylogenetic profiles have been discussed at length [35,36]. While shotgun metagenomics eliminates biases due to particular PCR primers, much work remains to be done in understanding biases introduced by sample preparation protocols, the reference database, and the nature of the reads recruited to the root node before linearity in phylogenetic profiles can be claimed. Since the shotgun metagenomics reads in our method were compared individually to a reference database for classification purposes, there is no need to correct for depth-of-sampling, as is typical for richness analysis (see e.g. [35]).

**Figure 8:**
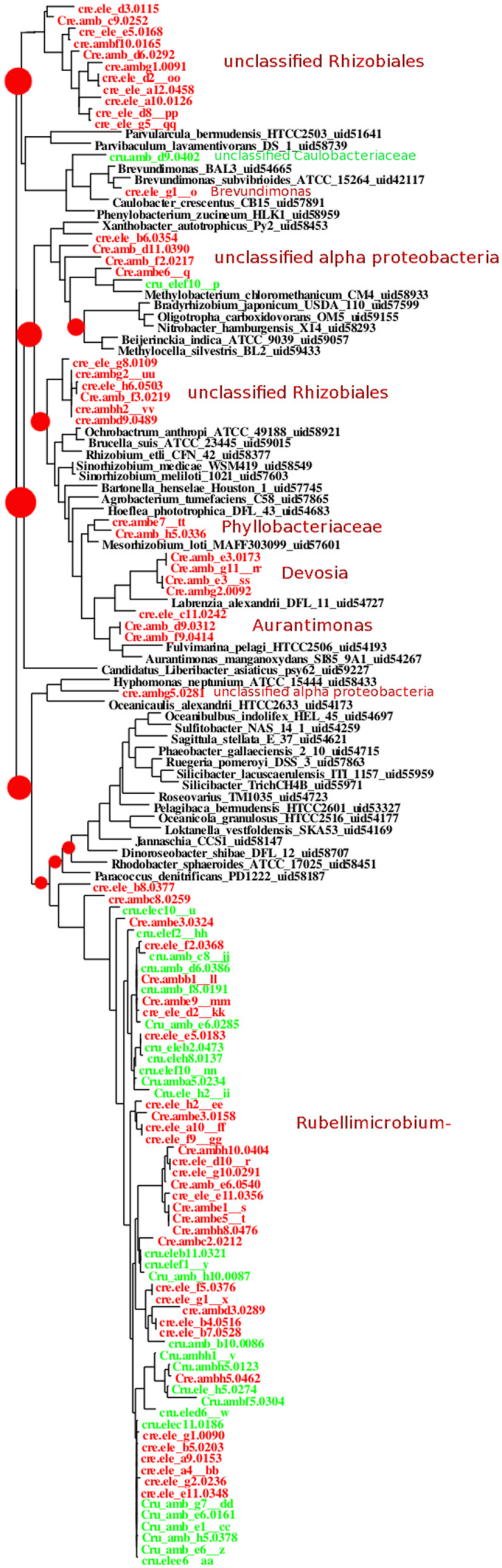
**Phylogeny of rhizobiales CREO and CRUST samples compared to reference database, using 16S sequences.** Maximum likelihood tree of full-length (black labels, reference genomes) and half-length 16S ribosomal sequences from Sanger sequence preparations of samples similar to the CREO (green labels) and CRUST (red labels) samples. The nine red dots at the nodes of the tree indicate the nine most populated nodes for the signature peptide analysis of the four samples represented in the CREO and CRUST samples, with an area proportional to the number of reads recruited. Labels to the right of the tree refer to assignments from the Baysian classifier at the ribosomal database project [53].

It seems likely to us that differences in the profiles from the different analysis methods arise from the way that each method treats ambiguous assignments, such as the transporter genes discussed in conjunction with Figure 1. The appropriateness of our choice to recruit to nodes, rather than to the leaves of the bacterial phylogeny, is supported by Figure 8, which shows the tree of reference genomes of a portion of the alpha proteobacteria, interspersed with the taxa from a 16S rRNA survey performed on samples from the CREO and CRUST sites. The preponderance of reads assigned to the deeply-branching nodes of this region of the tree, rather than nodes near the leaves, is well-supported by the 16S data, because genus-level matches to organisms in our reference database are not present. The resolution of phylogenetic placement is limited by the particular choice of organisms sequenced and not the phylogenetic resolution of the database of reference genomes, and our method correctly conveys this fact.

The soil bacterium *Rubellimicrobium mesophilum*, which was first isolated in Korean soil [37] appears in both the CREO and CRUST soil samples, yet is not in our reference database of completed genomes. We expect greater precision in phylogenetic classification as the reference database of completed genomes is expanded to include more examples of soil bacteria. Even though one of the environmental microbes identified in Figure 8 is likely present in some abundance in both CREO and CRUST, it is quite possible that another sample, taken only a few centimeters away, would show a different set of organisms [38].

Another encouraging aspect of the analysis of the soil metagenomics data is that our signatures matched roughly the same proportion of 375 base-pair soil metagenomics reads as MG-RAST with an E-value cutoff of 10-^10^, and they did so in a manner consistent with the signature’s appearance in the database of reference genomes. This is in contrast to the behavior of our method when using *k* = 8, described above, which indicates that signature peptides observed across a family of reference genomes are also valid signatures for identifying family members residing in the soils and not present in databases.

### Functional profiling

By selecting signature peptides that occur in at least two genera in our reference database, we have already selected a sub-set of signatures likely to have relevance to organisms not in the reference dataset. We therefore start with our list of 20 million signature peptides and assign a function to each signature peptide by searching a database of functionally-annotated genes from across the bacterial phylogeny. We chose the SEED database [39-41] as our source of functionally-annotated genes. Approximately two-thirds of the 20 million orthogenomic signature peptides were thus assigned a functional category (in this case a SEED subsystem) in addition to their phylogenetic classification. Some proteins, and therefore the signature peptides associated with those proteins, appear in more than one SEED subsystem. When this occurs, each SEED subsystem is assigned a fraction of a count such that each read is ascribed equal weight in assigning functional percentages, as described in Methods section, below.

**Figure 9:**
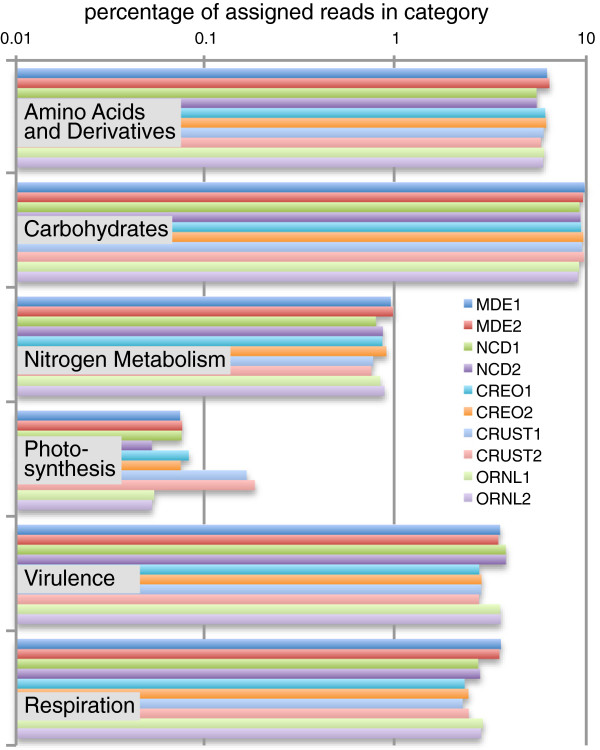
**Functional profile of metagenomic samples.** The functional assignments across the ten samples are broken down according to the highest level SEED categories, shown for six of 28 categories.

In the version of SEED we use, there are 1088 subsystems, which roll up hierarchically into two higher levels. Functional assignments for six of the 28 highest level SEED functional classifications are presented in Figure 9. The most striking aspect of this plot is the cross-sample consistency of the results among the different locations. The categories ‘amino acids and derivatives’ and ‘carbohydrates’ are functional processes that must be carried out by all bacteria, and contain numerous highly conserved genes; it is reasonable that the relative changes across the samples average only a few percent. Four of the categories shown, ‘nitrogen metabolism’, ‘photosynthesis’, ‘virulence’, and ‘respiration’, involve specialization and are carried out in different ways (and sometimes not at all) across the bacterial kingdom, so the larger differences seen between locations for these categories are also reasonable. Nitrogen metabolism is elevated in the estuary samples and suppressed in the desert crust. Photosynthesis is elevated 3-fold in the crust and suppressed in the ORNL samples. Virulence and respiration are both under-represented in the desert. Complete counts for all SEED categories are provided in Additional file 6.

### Ecosystem similarity

**Figure 10:**
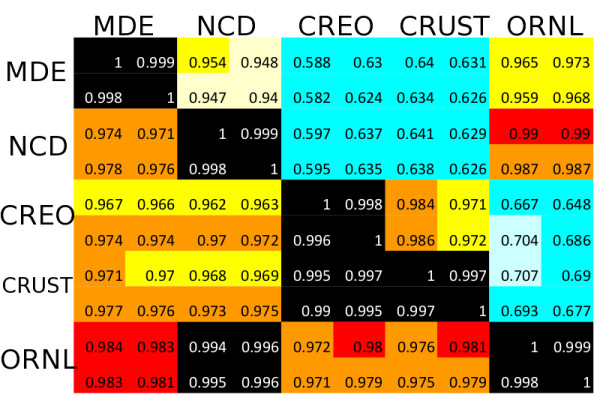
**Phylogenetic and functional similarity.** The normalized dot product (correlation) of phylogeny (upper right) and functional (lower left) profiles across the ten sites, defined by the number of reads assigned to each of the 402 nodes on the phylogenetic tree, or the 1088 SEED subsystems. For the phylogeny vectors, the root node was eliminated before computing the normalized dot product.

Both the phylogenetic profiles (Figure 7) and functional profiles (Figure 9) of the ten environmental samples are roll-ups of much larger vectors, with 402 elements for the phylogeny and 1088 elements for the function. Although many types of similarity metrics could be constructed from these profiles, we display simple normalized dot products in Figure 10, with the diagonal showing the identity of each sample with itself, the upper right triangle showing phylogenetic similarity among sites, and the lower left triangle showing functional similarity among sites. One striking feature evident in this figure is the repeatability between all five pairs of field replicates, with dot products of 0.999 for the phylogenetic profiles and 0.998 for the functional pressures. If we take one minus the dot product as a distance metric between sites, we can compute a dynamic range with this metric of 0.4 / 0.001, or 400, indicating significant information content can be extracted from each of the profiles, and that samples that are ‘representative’ of an ecosystem can be acquired and compared.

Further evidence of our signature peptide-based profile’s ability to highlight similarities and differences between ecosystems are that the two desert sites and the two forest sites are more similar to each other than desert is to forest, or to either of the estuary sediments. Also noteworthy is that the two desert sites are more similar to each other when compared by the function-based distance than the phylogeny-based distance.

Construction and interpretation of distance metrics is complex, and extracting ecologically meaningful insights from the phylogenetic and functional profiles will require both more samples and further analysis. Nevertheless, it is clear that the signature-peptide-based analysis can identify both commonalities and differences in both phylogenetic and functional attributes between ecosystem types.

### Functional specificity

**Figure 11:**
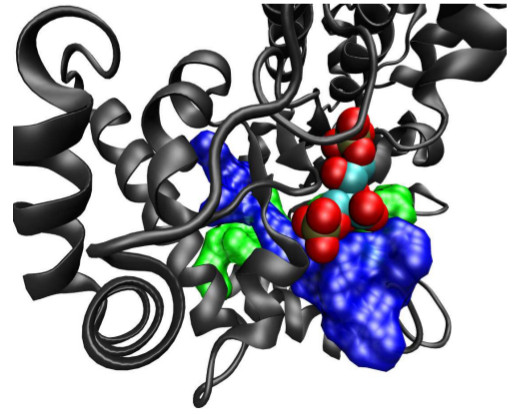
**Visualizing two root-level signature peptides in the enzyme RuBisCO.** Two root-level signature peptides (green and blue surfaces) correspond to regions of the protein which cross each other at an angle to form the bottom of a hydrophobic pocket where the substrate analog inhibitor 2,2-carboxyarabinitol-1,5-bisphosphate (spheres) binds. Residues in the signature peptides interact with the substrate, but also with each other. The former interactions contribute to substrate specificity, while the latter contribute to stability. Structure coordinates from PDB entry 1WDD.

Peptide signature analysis appears to work because the constraints of protein folding and function have sufficiently restricted the solution-space for most genes [7]. The handful of root-level signatures that we have viewed as 3-dimensional structures are consistent with this idea, with root-level signatures found lining ligand-binding pockets or other functionally-constrained sites near the active sites of enzymes where they interact with small molecules (and in some cases with each other, see Figure 11). Because a typical separation of genera near the leaves of our tree is ~ 10% amino acid divergence, signatures from nodes close to the leaves of the phylogenetic tree appear little different from randomly-selected non-signature fragments, but grow increasingly distinct near the root, where functional constraints predominate. For example, the pattern, GGxRxGEME is present in essentially every eukaryotic, archaeal, and bacteria RNA polymerase, and nothing else. When all of the 10-mers that overlap with this pattern are enumerated and placed on the phylogenetic tree, it is not only possible to classify divergent organisms, but also to provide insight into mechanistic differences in how various proteins function.

To compare the fidelity of functional assignments made by signature peptides and BLAST, we took the 2200 MDE fragments identified as part of the RNA polymerase (alpha, beta, beta-prime, gamma, omega, and delta subunits) and ran them against NCBI’s NR database. Upon reading them into MEGAN for SEED analysis, approximately half the reads were not assigned to any SEED subsystem, half to the RNA polymerase subsystem, and only one read assigned to a different SEED subsystem (virulence).

## Discussion

We have shown how finding fragments of DNA reads that code for proteins can be reduced to the simple and rapid operations of 6-frame translation followed by *k*-mer matching to signature peptides. Matching is intrinsically simpler than construction of local alignments, and many implementations of fast matching algorithms such as hash maps exist. Once the set of matches is obtained, assignment of phylogeny can be accomplished by a graph operation (greatest common descendant) that is fast compared with the translation and match steps. Any classification scheme that associates sequences with class can be subsumed by our method and the results counted; we have demonstrated functional classification with SEED, but other schemes such as KEGG, GO, Pfam, or any number of specialized classification schemes could have been used as well. Speeds of classification are such that a typical desktop computer running our analysis could easily keep up with the output of a next-gen DNA sequencer.

The perceived importance and practical difficulties of assigning phylogeny and function to metagenomic reads have spurred a significant amount of recent work exploring methods to reduce the size of the database against which local alignments must be performed as well as methods to estimate the appropriate specificity with which to assign a particular metagenomic read. We discuss here how our method relates to published work in these areas, explain how we tested specificity and sensitivity, and discuss extensibility of our method.

### Speed compared to existing methods

The 250,000-fold speedup we obtain in comparison to running BLASTX against the NR database results as the result of three strategies: speeding up local alignment as much as possible, reducing the size of the reference database as much as possible, and pre-computing the phylogenetic relationships as much as possible. The first two of these strategies have been previously implemented by other analysis tools in one form or another.

It is possible to speed up homology searches with respect to BLAST by seeding the search with exact matches that are longer than the BLAST default of 3 residues. BLAT [42] uses 4-residue seeds and reports a 50-fold speedup with respect to BLASTX, while RAPSearch2 [43] uses 6-residue seeds and reports as 20–90 fold speedup when searching the NR database with little or no reduction in sensitivity or specificity. Figure 1 shows that the number of seeds that need to be considered drops by a factor of 5000 when comparing 6-mer to trimer seeds. It also shows that, depending on phylogenetic distance, between 90 and 99% of the matches identified by 6-mers are noise, in the sense of the term described in Figure 1. One way of thinking about our work is that we have extended the seed length from 3 residues to its practical limit of 10 residues, beyond which sensitivity drops off markedly. At that length the seed is the alignment, eliminating the need for further explicit local alignments. Even when running BLASTX against a database with the same 403 complete bacterial genomes we used, and assuming the upper-end 100-fold speedup described in [43], our method still exhibits a 160-fold higher throughput.

Another strategy for reducing search-space is to differentiate homologous protein matches with the synonymous nucleotides, typically at the third position of codons. As we observed with the synthetic data shown in Figure 6, this strategy increases the rate of BLAST search by approximately fifty, without a great cost in sensitivity. However, using this strategy does seem to increase the extent to which the presence of a near-neighbor in the reference database influences the sensitivity with which reads are assigned. Because codon usage can be discerned with relatively short genomic fragments without searching for amino acid homology, it is possible to do ‘compositional binning’ to provide a phylogenetic classification in the absence of a gene-homology search [44-46]. Given the desire of most researchers to exploit the observed homology of proteins from different organisms in their analysis, and the significant unpredictability of nucleotide patterns across the phylogenetic tree, it seems unlikely that these methods can be significantly improved. It is certainly possible to identify ‘signature oligonucleotides’ in the same manner as we have done for signature peptides, and one can easily imagine applications, such as looking for known pathogens, where nucleotide signatures will be valuable.

Analysis of the synthetic data shown in Figure 6 showed that the ability of BLASTX to assign the genomic data from soil bacteria did not degrade much when utilizing the same database of organisms that we used to generate our signature list, although throughput increased by approximately 60-fold. The results of the 16S comparison to completed genomes in Figure 8 provide a good indication as to why; the database is not well representative of soil bacteria. This observation supports our decision to place a minimum phylogenetic distance across which a peptide has to be observed in order to qualify as a signature peptide. Similar reasoning was behind efforts to seek out phylogenetically divergent bacteria for sequencing, in order to produce a Genomic Encyclopedia of Bacteria and Archaea [47]. Although this effort was just getting under way as our work began, we incorporated the forty genera of bacteria that were only available from this source into our reference set of genomes, and these organisms are indicated in Additional file 7.

Search-space can also be restricted by performing the phylogenetic classification within each protein family [48] or utilizing only a sub-set of ‘housekeeping genes’ for phylogenetic classification [20]. Neither of these two methods is particular rapid at identifying the subset of genes corresponding to a particular protein family. Because the signature peptides carry a functional assignment as well as a phylogenetic one, it is possible to perform a more detailed phylogenetic analysis on, for example, only the RNA polymerase genes; we showed that there were 2200 such fragments in the pair of MDE samples. Once this down-select is performed, it is possible to use complex tree-building algorithms, curated alignments, and assess quality scores of the metagenomic reads to obtain a detailed understanding of how the organisms in the metagenomic sample relate to those in another sample or the reference database. With the signature peptides, however, it is also possible to utilize past performance of particular signatures to screen for those which provide reliable phylogenetic assignments.

### Sensitivity and specificity

Our strategies of using a length of 10 residues for matches and using a minimum phylogenetic distance cutoff, thereby eliminating need for a local alignment step and reducing the size of the signature list by 95%, appear to be novel and require demonstration that they do not adversely impact specificity and sensitivity. Testing for specificity is best done with two divergent and well-annotated genomes. We chose *E. coli* and *B. subtilis* and the results are provided in Figure 3 and Additional file 1. Given the practical value of even 5-mer peptides in rapidly identifying particular genes from thousands of complete genomes (data not shown), it is perhaps not surprising that 10-mer exact matches exhibit a great specificity. A test of the specificity of signature peptides on metagenomics data showed that they disagreed with BLASTX on ~350 base pair reads in only one case out of 2200 for the case of the RNA polymerase proteins.

The question of specificity of phylogenetic placement is somewhat ill-posed, and arguably depends on the type of data analyzed and the purpose of the experiment. By comparing our methods to three other representative methods in Figure 6, we have demonstrated they are comparable to one another in their ability to accurately place reads from a novel organism on an existing phylogeny. In Figure 10, we propose a different metric for the specificity of placement of metagenomic reads: How well can the phylogeny and function count vectors differentiate among metagenomes (signal) in comparison to their repeatability for replicates (noise)? Figure 10 demonstrates a signal-to-noise ratio of 400 for the phylogeny vector and 30 for the SEED function vector. We did not perform this calculation for alternative methods, but it appears the signature peptides will be valuable when used in this manner.

Perhaps more surprising, and definitely more subtle, is that the sensitivity of our method is comparable to BLASTX against NR, as demonstrated with synthetic data from organisms novel to the set of reference genomes in Figure 6, and metagenomics data in Table1. The modest decrease in sensitivity of approximately 25 percent is largely explained by the observation from Figure 3 that 20% of the genes in a typical genome do not contain 10-mers matching to another genome in our reference set of genomes. That the decreased sensitivity is due to the discarding of more variable (in the sense of gene inventory) proteins is supported by the relatively small variation of sensitivity observed among the four novel organisms presented in Figure 6, especially in comparison to BLASTN. Some understanding of why 10-mer exact matches have a high probability of matching a gene from a divergent organism when a cursory glance at a pairwise sequence alignment suggests such matches would be rare is provided by Figure 2. Genes that contain a signature peptide tend to have more than one scattered throughout the gene and typically match to multiple organisms. Once all the pairwise comparisons are made across hundreds of reference genomes, a pretty thorough sampling of possibility space is obtained. Support for the idea that possibility-space is well-sampled is found in the observation from the FACE data results that 83% of matching reads contained multiple signature peptides. Figure 11 suggests an explanation for why so many genes contain signature peptides might be that root-level signature peptides preferentially lie near the active sites of enzymes, where only a limited set of amino acid sequences are sufficiently adept at interacting with small molecules for the gene to propagate, along the lines suggested in [7].

The impact of sequencing errors on our method is relatively straightforward to understand. Because our method requires an exact match to an amino acid 10-mer, and because the number of signatures (3x10^7^) is so much smaller than possibility space (10^13^), the dominant effect of introducing errors is a simple decrease in sensitivity given by the likelihood of the sequencing error occurring within all of the signatures in the read.

### Extensibility

The software package BLAST was released over twenty years ago [5] at a time when sequence databases were much smaller and simple identification of sequence homology was quite valuable. Since that time, both the implementation and interpretation of BLAST has undergone significant evolution, the size of reference databases has increased by many orders of magnitude, and the types of questions asked of sequencing projects has changed significantly. Indeed, it would be possible to implement a process quite similar to our own within the space of allowed options of BLAST and a modest amount of additional post-processing. Nevertheless, a shift from interpretation of similarity scores of local alignments to phylogenetic identification of significant matches not only significantly speeds the analysis process, but makes feasible several new types of analyses. We explore some of them here.

Curation and refinement of signature peptide lists, whether with additional layers of algorithms, with manual intervention, or both, is certainly possible and attractive. The list of reference genomes could be expanded in resolution (to the species level) and extended to the other kingdoms of life. Signature peptides could be identified that are likely to be indicative of leaves on the tree, rather than nodes. Signatures derived from mobile elements such as plasmids could be identified as such and indicated as an attribute of the respective signature peptides. The 22% of the reads with conflicting phylogenetic signatures can be analyzed further to reassign signatures on the tree and thereby increase the specificity of classification where appropriate. The network of genes sharing a signature peptide can be subjected to analysis aimed at simplifying the graph structure by associating signature peptides with domains rather than with entire proteins.

Using the SEED functional classification scheme allowed us to compare broad categories of protein function, but much more work is needed on functional signature classifications that efficiently capture variances in real data while preserving connections with small molecules and pathways. Additional algorithms could be derived to extract 'niche' signatures from multiple metagenomics samples or sequencing data which is derived from a small group of organisms which cannot readily be separated. This information could be combined with curated databases of protein families and co-localization of signature peptides on either reference genomes or long-read metagenomics data. While much of the above is being explored within the context of BLAST and hidden Markov models, the signature-peptide formalism naturally lends itself to extension in areas such as these.

A recent example that exploits the ability of signature-based analysis to distinguish inheritance from horizontally transfer was used to shed insight on the nature of virulence in enteric bacteria [49]. This ability of signature-based methods to be embedded in more sophisticated algorithms, plus our method’s local signatures and large phylogenetic radius of convergence, make the method particularly well-suited to a wide range of currently intractable sequence analysis problems.

Finally, signature peptides may be useful as physical objects in addition to being search terms. Peptide 10-mers are suitable to use as antigens for developing immunochemical assays of microbial community dynamics, though processing may be needed to make the corresponding protein fragments accessible to antibody binding. The amino-acid composition and positional dependence in signature peptides becomes significantly different from random selections of peptides from the genomes involved as the signature placement nears the root (not shown). The nature of these differences suggests that some root-level signature peptides may play a role in formation of hydrophobic pockets that bind small molecules. If true, signature peptides may be the minimal functional units that form the starting point for evolution, and may also be useful as fragments that are diagnostic of possible protein interactions with a given small molecule.

## Conclusions

We have demonstrated that metagenomics reads can be accurately assigned both phylogeny and function entirely by a matching to a sorted list of 10-mer signature peptides. We also developed and utilized algorithms to identify the signature peptides, to assign individual signatures to nodes of a phylogenetic tree and categories of protein function, and to assign individual metagenomic reads both a phylogeny and function. Our software runs on a desktop-class computer, identifying protein fragments and classifying them for phylogeny and function at a rate of ~6.6 Gbp per hour on a single core, over 250,000 times the throughput of BLASTX run against the NR database [40] and about twice the rate of current sequencer output. We demonstrated our process on shotgun metagenomic reads on soil samples from five diverse ecological sites, with two field replicates from each location. We observed a sensitivity comparable to analysis performed at MG-RAST with an E-value cutoff of 10-^10^, a repeatability between field replicates of better than 99.9%, and a signal to noise ratio for distinguishing ecosystems of approximately 400. Having such a rapid alternative to conventional homology searches for phylogenetic and functional classification of short reads of DNA seems likely to impact bioinformatic applications beyond its immediate application to metagenome analysis.

## Methods

### Sample collection and sequencing

Sample collection and preparation was carried out as previously described [34,50]. Sequencing was carried out on a 454 Genome Sequencer Titanium system at the LANL Joint Genome Institute. 454 sequencing is known to suffer from spurious near-duplication of reads [51,52]. We used the program 454ReplicatesFilter [51] v20090611 with default parameters to identify and remove on average 12% (ranging between 4% and 21% per sample) of reads. 16S rRNA sequencing was done on an Applied Biosystems 3730xl instrument and analysis was performed at the Ribosomal Database Project website using RDP release 10 update 24 [53].

### Reference genomes and phylogenetic tree calculation

A list of reference bacterial genomes is included as Additional file 7. Bacterial genomes were downloaded from NCBI (completed) and JGI (draft) in June of 2009. A phylogenetic tree was calculated based on the concatenated amino acid sequences of the beta and beta-prime subunits of the RNA polymerase. An initial multiple sequence alignment was calculated using MUSCLE [23], followed by iterative manual curation of the alignment with BioEdit [54] based on the known three-dimensional structure, and tree building with a maximum likelihood method employing a minimal model of protein functional pressure (RIND [55] and WEIGHBOR [56]). We placed the root of the tree at the long branch connecting gram-positive and gram-negative bacteria, in accord with current understanding of bacterial evolution [57]. the resulting tree (Additional file 2 and Additional file 3) compares well to those in the literature [58] and with 16S rRNA-based trees; it disagrees from the less-detailed NCBI taxonomy (where available) in only a handful of cases. Using this tree, the number of genomes was manually pruned from the total available genomes at that time to a reference set of 403 genomes that were separated from one another by a minimum evolutionary distance of 0.015 as calculated by RIND. This distance corresponds approximately to the distinctions conferred by genus names. Subsequent analysis used only the connectivity, not the distances in this tree.

### Signature production

Calculation of signature peptides was carried out in version 0.8 of a program suite consisting of Python 2 and Java 1.6 code we call Sequedex. Biopython [59] v1.54 and Forester [60] v0.970 were used to manipulate phylogenetic data in PhyloXML. Protein sequences from genomes and putative peptide fragments from metagenomes were treated as documents, broken into overlapping *k*-mer terms, and indexed by Lucene [61] v2.4.1 with a custom *k*-mer tokenizer that allowed *k* to be specified at run time.

**Figure 12:**
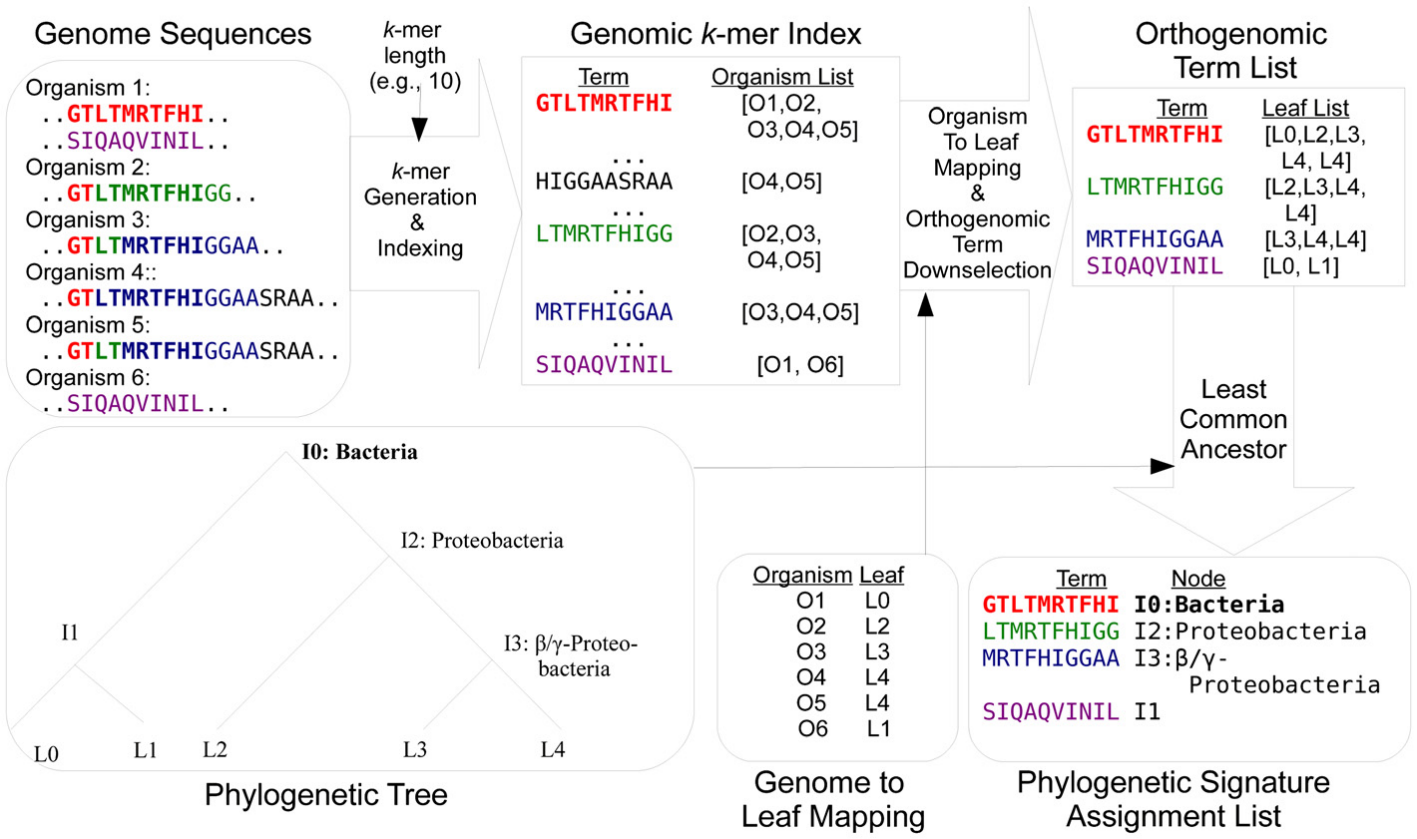
**The signature production process.** Approximately 400 million overlapping 10-mers from the 403 bacterial reference genomic sequences are enumerated and collated into a genomic *k*-mer index. The 5% of this list that appears in multiple genera of bacterial reference genomes are collected, together with the list of leaves (taxa) which contain the signature. Using our inferred phylogeny of reference genomes (provided as Additional file 2, Additional file 3), we use the least common ancestor algorithm to assign the signature to the most specific node that covers all observations of the 10-mer.

Figure 12 shows the process of signature generation. *k*-mer terms from every protein sequence in each of the reference genomes were merged into a single index. Terms that did not appear in more than one leaf were discarded, leaving only orthogenomic terms. Each orthogenomic term was assigned to the internal node on the phylogenetic tree that was the least common ancestor of the leaf nodes in which the term appeared. Terms from a single organism thus can be associated with any of the internal nodes along the path from the leaf node containing the organism to the root of the tree.

### Production of putative protein fragments

**Figure 13:**
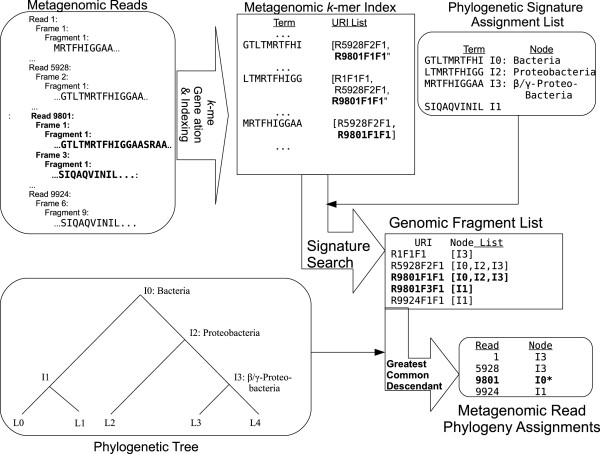
**The metagenomic read analysis process.** Metagenomic reads are translated in the 6 possible reading frames as peptides and indexed as 10-mers of amino acids. This index is searched for phylogenetic signatures and the node assignments of the signatures are collated per read. Reads are assigned to nodes on the phylogenetic tree at the most specific node for which there is consistent evidence via the greatest common descendant algorithm.

Figure 13 shows the process of metagenome phylogenetic analysis. Six-frame translation to amino-acid space was carried out with the EMBOSS [62] utility transeq, with default parameters. Each frame was broken into putative peptide fragments (starting with either the upstream read boundary or the first residue after a stop codon, ending with either a stop codon or the downstream read boundary), subject to a minimum length restriction of 15 residues. This length restriction served to decrease the number of putatitve peptides to be searched without a serious decrease in sensitivity. Ambiguous residues were treated as stop codons.

### Matching and phylogenetic assignment of reads

Each putative protein fragment was broken into overlapping 10-mer peptides which were then matched against the list of signature peptides. Fragments that contained one or more signatures were thereby identified and lists of node assignments from the signatures were built up for each read. In most cases, the list of node assignments was a subset of the internal nodes along a single path from the leaf to the root (monophyletic). In this case, the read is assigned to the most specific node found (the node that is farthest from the root). The possibility exists, however, of a read arising from an organism whose protein domain inventory differs from those in the reference genomes in such a way that nodes from more than one path from leaf to root will be found (non-monophyletic). In this case, we assign the read to the node that is the least common ancestor of those nodes that are farthest from the root. Phylogeny assignment calculations are faster than the translation and matching steps.

For all the analysis presented here, matching was done via the index structure produced by Sequedex and Lucene. However, since none of the analysis relied on the additional information in the index beyond which signature peptides matched and since indexing was the slowest step in the process (~0.2 Gbp/hr) we wrote code to do the search without having to make an index which we call Sequescan. Sequescan reproduces the results of the index-based Sequedex, but it processes data (as of version 0.1) at a rate of approximately 6.6 Gbp/hr (from FASTA file to classification) on a single core of a Intel Core i7 machine with a fixed memory requirement of <6 GB. The processing rate per CPU and memory usage of Sequescan seems to be approximately independent of read length and is independent of number of processes up to at least 4. We are writing a multi-threaded version of Sequescan that we expect to be available at time of publication for free download as listed below.

### Generation of synthetic data

Synthetic data were created from complete genomes of *Shigella flexneri* 2a and *Dehalogenomonas lykanthroporepellens* BL DC 9 uid48131 as well as the four draft genomes used to generate Figure 6, using MetaSim v.95 [63] with no error model, no paired ends, and fixed read length.

### Functional assignment of reads

Functional assignment of metagenomic reads is done by collating the functional assignments of the matching signatures. To this end, we looked for signatures that could be found in sequences from the 1088 subsystems of the SEED database [39-41], downloaded in January of 2010. Signatures were assigned to all subsystems that matched. On average, 69% of bacterial reads in any sample were assigned to one or more SEED subsystems. A single read is assigned to n subsystems by computing the union over all reading frames of the intersection of subsystems associated with each reading frame for which orthogenomic signatures were found. Each assigned subsystem is then allotted *1/n* counts for this read. For Figure 9, SEED subsystems were hierarchically grouped into the 28 high-level categories found in the SEED file 'subsystems2role'.

### Availability

Free software to produce the phylogenetic and functional profiles described here for arbitrary metagenomics or synthetic data sets will be made available at http://sequedex.lanl.gov.

## Competing interests

Los Alamos National Security, LLC (operator of Los Alamos National Laboratory) has a patent pending on ideas related to those described in this manuscript and is actively seeking partnerships to commercialize a user-friendly implementation of this methodology with extended functionality.

## Authors’ contributions

N.H. and B.M conceived the study; J.B. proposed the method and designed the indexing and analysis algorithms; M.W. provided the signature concept; J.B. and J.C. wrote the software; J.C. performed data wrangling and ran the final analyses; B.M. did the phylogenetic and exploratory data analysis; N.H. and J.B. performed statistical analyses; C.K. selected the sites, collected and prepared samples, and supervised sequencing; G.X. and J.C. performed the analyses by alternate methods; and B.M. and J.B. wrote the paper. All authors discussed the results and commented on the manuscript. All authors read and approved the final manuscript.

## Supplementary Material

Additional file 1 ** List of 10-mer matches. This file contains a tabdelimited text table of 10-mer or longer matches between E.** coli and Bacillus subtilis, with the annotation and amino acid sequence of the genes containing the match. Click here for file

Additional file 2 ** Phylogenetic tree with node numbers.** This file contains a pdf file of the phylogenetic tree of the 403 reference bacterial genomes used to assign phylogeny to both signatures and metagenomic reads. Node numbers are provide for use in Additional file 5. Click here for file

Additional file 3 ** Phylogenetic tree with node numbers.**This file contains a phyloxml file of the phylogenetic tree of the 403 reference bacterial genomes used to assign phylogeny to both signatures and metagenomic reads. Node numbers are provide for use in Additional file 5. Click here for file

Additional file 4 **Synthetic data produced from draft genomes of four soil bacteria.** This file contains a zip file of the 16 synthetic data sets used to compare sensitivity, specificity, and throughput of our method to three types of BLAST-based methods. (TAR 6400 kb) Click here for file

Additional file 5 **Phylogenetic profile of metagenomic samples.** This file contains a tab-delimited text table of the number of reads assigned to each node on the phylogenetic tree for each sample. Node numbers refer to the phylogenetic tree shown in Additional file 1 and Additional file 2. (TXT 13 kb) Click here for file

Additional file 6 **Functional profile of metagenomic samples.** This file contains a tab-delimited text table of the number of reads assigned to each of the 1088 SEED categories, for each sample. (TXT 91 kb) Click here for file

Additional file 7 **Reference genomes.** This file contains a tab-delimited text table of reference genomes used, with source for each. (TXT 32 kb) Click here for file
