# Supplementary figures and images for: Rapid phylogenetic and functional classification of short genomic fragments with signature peptides
Source: BMC Res Notes. 2012 Aug 28;5:460. doi: 10.1186/1756-0500-5-460 (PMC3772700; doi:10.1186/1756-0500-5-460)

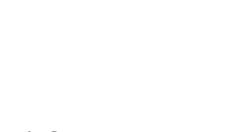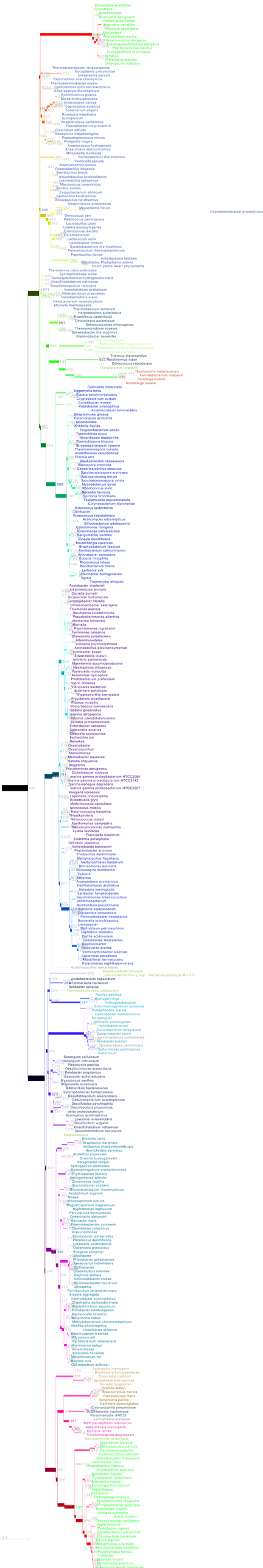

Supplement: Additional file 2 — Phylogenetic tree with node numbers. This file contains a pdf file of the phylogenetic tree of the 403 reference bacterial genomes used to assign phylogeny to both signatures and metagenomic reads. Node numbers are provide for use in Additional file 5. [file 1756-0500-5-460-S2.pdf]
